# SNMP1 Contributes to Sensitive Pheromone and Plant Volatile Detection in the Locust Olfactory System

**DOI:** 10.3390/ijms27188291

**Published:** 2026-09-17

**Authors:** Joris Lehmann, Jürgen Krieger, Joerg Fleischer

**Affiliations:** Department of Animal Physiology, Institute of Biology/Zoology, Martin Luther University Halle-Wittenberg, 06120 Halle (Saale), Germany; joris.lehmann@zoologie.uni-halle.de (J.L.); juergen.krieger@zoologie.uni-halle.de (J.K.)

**Keywords:** insect olfaction, sensory neuron membrane protein 1, *Schistocerca gregaria*, antenna, volatile organic compounds, plant odorants, electroantennogram

## Abstract

In holometabolous insects, such as flies and moths, the sensory neuron membrane protein 1 (SNMP1) has been primarily linked to the olfactory detection of long-chain aliphatic pheromones, but whether it plays an important role in receiving other chemical cues is largely unknown. Here, comparing electroantennogram (EAG) recordings of wild-type (WT) and SNMP1-deficient (SNMP1^−/−^) hemimetabolous desert locusts (*Schistocerca gregaria*), we found that mutant animals showed significantly reduced responses to several pheromones and plant odorants belonging to different classes of aromatic and aliphatic substances. Our findings show that SNMP1 contributes more broadly to the detection of various chemicals, such as benzenoids, aliphatic aldehydes, and non-aromatic cyclic substances. Importantly, the decrease in antennal reactivity of SNMP1 mutants to certain chemicals was higher at lower concentrations of these substances, suggesting the relevance of SNMP1 for the sensitivity of olfactory detection. Moreover, behavioral experiments with WT and SNMP1^−/−^ animals showed that SNMP1 is crucial for the attraction of desert locusts by food plant scents. To examine whether diminished olfactory responses in SNMP1^−/−^ animals were due to reduced expression of odorant receptors (ORs), transcriptional levels of selected OR types were scrutinized by quantitative PCR (qPCR). In these approaches, no significant reduction in the expression of the ORs was found in SNMP1^−/−^ locusts, suggesting that the compromised responsiveness to certain substances is presumably not based on impaired receptor expression. Overall, our findings reveal that SNMP1 is critical for olfactory sensitivity in locusts and expand the current view of SNMP1 from a protein particularly essential for receiving aliphatic pheromones to a more general olfactory element important for proper sensing of a broader range of chemical cues, including plant odorants.

## 1. Introduction

For many insects, the recognition of pheromones as well as plant-derived volatiles is of crucial importance, as they indicate suitable mating partners, promising food sources, and appropriate egg-laying sites, among other things. To receive general odorants and pheromones, the antennae of insects are equipped with cuticular structures called sensilla that harbor olfactory sensory neurons (OSNs). In locusts, OSNs are housed in three morphologically distinct olfactory sensilla types, i.e., sensilla coeloconica, sensilla trichodea, and sensilla basiconica [1]. The dendritic membrane of insect OSNs is endowed with olfactory receptor proteins that usually belong to the families of odorant receptors (ORs) and ionotropic receptors (IRs) [2,3,4,5,6]. OR-positive OSNs are characterized by the co-expression of the odorant receptor co-receptor ORCO [7] that forms a heteromeric cation channel with ligand-binding OR proteins [8,9]. Given subsets of OR-positive OSNs are also endowed with the so-called “sensory neuron membrane protein 1” (SNMP1), a protein comprising two transmembrane and a large extracellular domain that was originally discovered in the membrane of the dendrites of pheromone-responsive OSNs of moths [10,11,12,13]. SNMP1 belongs to the large and diverse CD36 protein family encompassing vertebrate and invertebrate members [12]. Some proteins of the CD36 family are considered to serve as transporters and receptors for lipids, including long-chain fatty acids, and lipoproteins [14,15,16]. Previous studies in holometabolous insects, namely *Drosophila* flies and moths, have demonstrated that in OSNs, SNMP1 is essential for sensitive reception of long-chain aliphatic pheromones involved in controlling sexual behaviors, such as cis-vaccenyl acetate (cVA) of *Drosophila melanogaster* [10,17] or (Z)-11-hexadecenal, and (Z)-9-hexadecenal in moths [18,19]. However, the precise role of SNMP1 in insect pheromone detection is still elusive. In this regard, it was found that the extracellular domain (ectodomain) of the SNMP1-related protein CD36 can bind long-chain pheromonal compounds [20]. Moreover, homology modeling based on X-ray crystal structures of a mammalian CD36 family member suggested that the ectodomain of *Drosophila melanogaster* SNMP1 might form a tunnel-like structure for funneling pheromonal compounds to the cognate pheromone-binding OR type in the dendritic membrane of OSNs [20].

In contrast to the holometabolous *Drosophila* fly and moth species, the function of SNMP1 has hardly been investigated in hemimetabolous insects, such as the desert locust *Schistocerca gregaria* (belonging to the order of Orthoptera), an economically and ecologically important insect pest dreaded for its capability to form huge swarms, devastating crops and natural vegetation in invaded areas of Africa and Western Asia [21,22,23]. In *S. gregaria*, SNMP1 is co-expressed with at least 33 of the known 119 OR types of this species [24,25]. Consequently, SNMP1 seems to play an exceptionally crucial role for olfactory signaling in this insect and might interplay with various ORs, thus contributing to the sensitive detection of a variety of chemicals. In contrast to other insects, in which SNMP1 serves the reception of long-chain pheromones, for desert locusts, pheromonal compounds with long carbon chains have not yet been described. Recently, we showed that in *S. gregaria*, SNMP1 is essential for the detection of phenylacetonitrile (PAN) [26], a pheromone associated with the reproductive behavior of desert locusts [27,28]. Importantly, PAN is a benzenoid aromatic compound lacking a long carbon chain, suggesting that locust SNMP1 may have a function in the detection of compounds structurally quite different from the long-chain, non-aromatic (aliphatic) pheromone substances of *Drosophila* flies and moths. In this context, it is interesting to note that further benzenoid aromatic substances have been reported to serve as pheromones in the desert locust, including acetophenone, veratrole (1,2-dimethoxybenzene), 4-vinylveratrole, and benzaldehyde [29,30,31]. Moreover, several non-aromatic pheromonal compounds with rather short or medium-length carbon chains have been described to influence aggregation behavior of *S. gregaria* nymphs, such as the aldehydes hexanal, octanal, nonanal, and decanal [32]. However, it is currently unknown if SNMP1 contributes to the detection of pheromones other than PAN in *S. gregaria*.

With respect to aromatic and non-aromatic pheromone compounds in *S. gregaria*, some of them, such as the benzenoid aromatics PAN, acetophenone, veratrole, 4-vinylveratrole, and benzaldehyde, as well as the aliphatic substances hexanal, octanal, and nonanal, are also found in plants [33,34,35,36,37,38,39]. This led us to speculate that SNMP1 might also be involved in the detection of plant substances, such as volatile organic compounds (VOCs). VOCs are extremely important for numerous insects, as these chemicals lure pollinators or repel herbivores, for example, by attracting predators or parasitoids through increased emissions during an herbivore attack [40,41,42,43]. VOCs are often lipophilic substances with a low molecular weight and high vapor pressures. More than 1700 VOCs have been identified in plant species that release them from leaves, flowers, fruits, stems or roots. According to their biosynthetic pathway, VOCs are frequently classified into three major groups: terpenoids, benzenoids/phenylpropanoids, and fatty acid derivatives [41]. With regard to the antennal detection of VOCs, it has been shown in *Drosophila melanogaster* via SNMP1 mutants that the function of SNMP1 is not confined to the detection of the pheromone cVA but is also required for the responses of a given subset of OSNs to the terpenoid farnesol [44], an attractive odorant of citrus fruits proposed to indicate appropriate oviposition sites to *Drosophila* females [45,46,47]. Moreover, behavioral studies with two hemimetabolous insects, the white-backed planthopper *Sogatella furcifera* and the aphid *Rhopalosiphum padi* (both belong to the order of Hemiptera), in which the expression of SNMP1 was knocked down by RNA interference (RNAi), have demonstrated that SNMP1 is crucial for seeking behavior and attraction to host plants [48,49]. Collectively, the previous results suggest that the role of SNMP1 in the insect olfactory system may extend far beyond the detection of pheromones. Against this background, we set out to examine the importance of SNMP1 in pheromone and plant odorant (VOC) recognition of the hemimetabolous desert locust. Towards this goal, we investigated whether the electrophysiological responses in the antenna to pheromones as well as various plant-derived volatiles depend on SNMP1, examining a range of aromatic and aliphatic compounds. To this end, we made use of a recently generated *S. gregaria* mutant line (SNMP1^−/−^) that lacks a functional SNMP1 [26], and compared its olfactory responsiveness to wild-type (WT) animals in electroantennography (EAG) recordings.

## 2. Results

### 2.1. The Detection of Several Aromatic Pheromone Compounds Is Impaired in SNMP1-Deficient Desert Locusts

**Figure 1:**
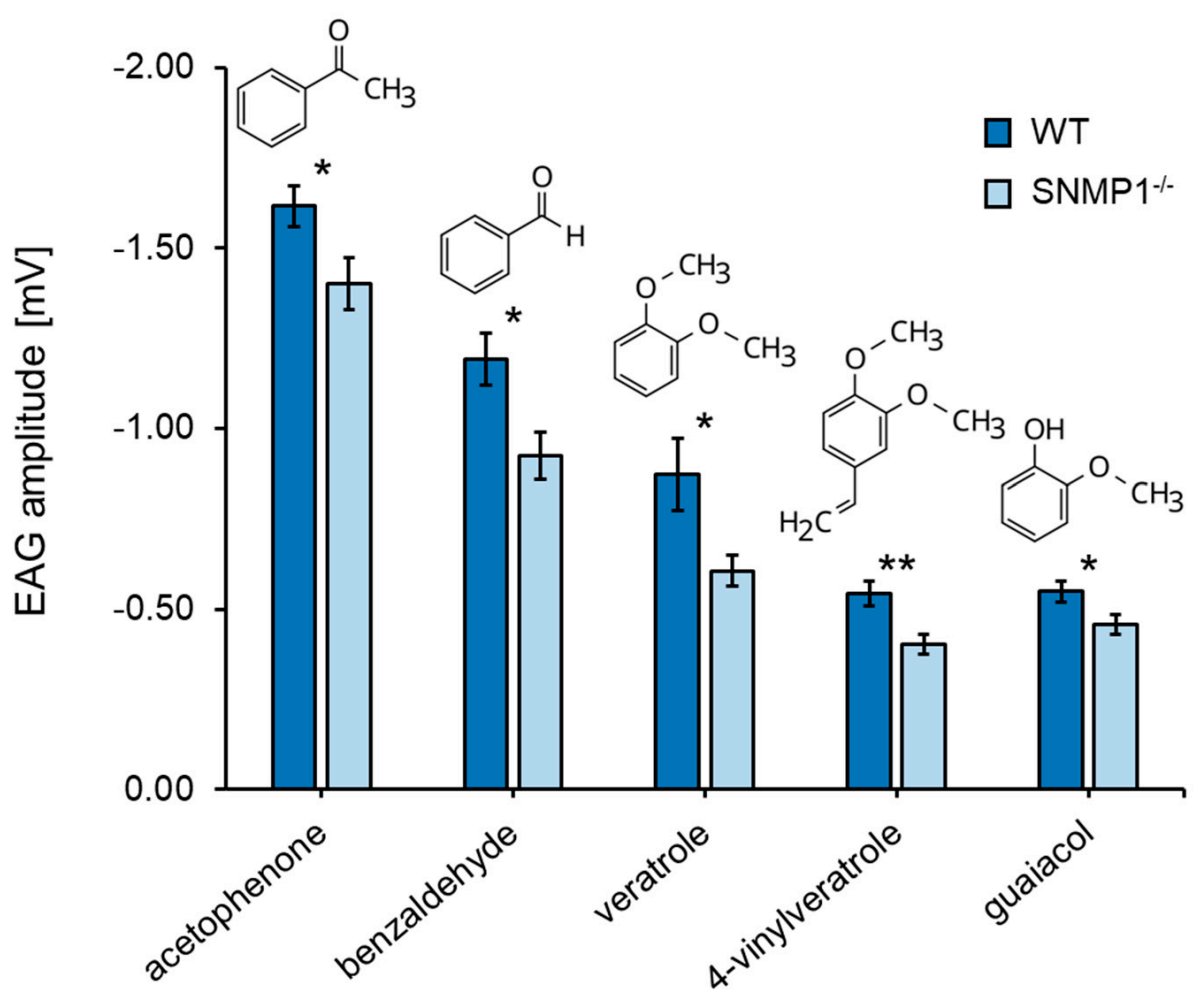
EAG responses to aromatic pheromones and semiochemicals released by desert locusts are significantly reduced in SNMP1 mutants. EAG responses were recorded from the right antenna of wild-type (WT; dark bars) and SNMP1-deficient (SNMP1^−/−^; light bars) males. Mean EAG amplitudes triggered by the indicated benzenoid substances in the antennae are shown. A total of 10–25 WT and 11–25 mutant antennae were analyzed for the above-mentioned substances. All compounds were tested at a dilution of 1:100 (in DMSO). The standard error of the mean (SEM) is given by error bars. Two-tailed *p*-values were calculated by unpaired *t*-tests (**, 0.001 ≤ *p* < 0.01; *, 0.01 ≤ *p* < 0.05).

To assess if the detection of aromatic pheromone compounds other than PAN relies on SNMP1 in *S. gregaria*, EAG recordings were performed using antennae of WT and SNMP1^−/−^ males. We first tested three aromatic pheromone compounds that accelerate maturation in immature male desert locusts, including benzaldehyde, 4-vinylveratrole, and veratrole [31]; the latter substance also controls egg laying in *S. gregaria* [29]. In addition, EAG recordings were conducted with the oviposition-eliciting aromatic pheromone acetophenone [29] and the aromatic fecal semiochemical guaiacol, which is considered to be part of an *S. gregaria* aggregation pheromone cocktail [30,50]. Testing of the five benzenoid substances at a dilution of 1:100 revealed that the electrical responses elicited in the antennae of males were significantly lower in SNMP1^−/−^ animals compared to WT conspecifics (Figure 1). More specifically, EAG amplitudes were reduced in the antennae of mutants by ~13% (acetophenone) to ~30% (veratrole) in comparison to WT males. Next, we also examined the antennal reactivity to the aromatic substance anisole. Although, to our knowledge, there is so far no clear indication that anisole functions as a pheromone in *S. gregaria* [31], it is released by mature male desert locusts [51]. At a dilution of 1:100, anisole induced significantly lower EAG responses in SNMP1^−/−^ animals compared to WT conspecifics (Figure S1). Thus, SNMP1 seems to be involved in the reception of the benzenoid compound anisole. Taken together, our findings indicate that SNMP1 is not only crucial for detecting the aromatic pheromone PAN [26], but also contributes to receiving a range of other benzenoid pheromone compounds and semiochemicals of the desert locust.

**Figure 2:**
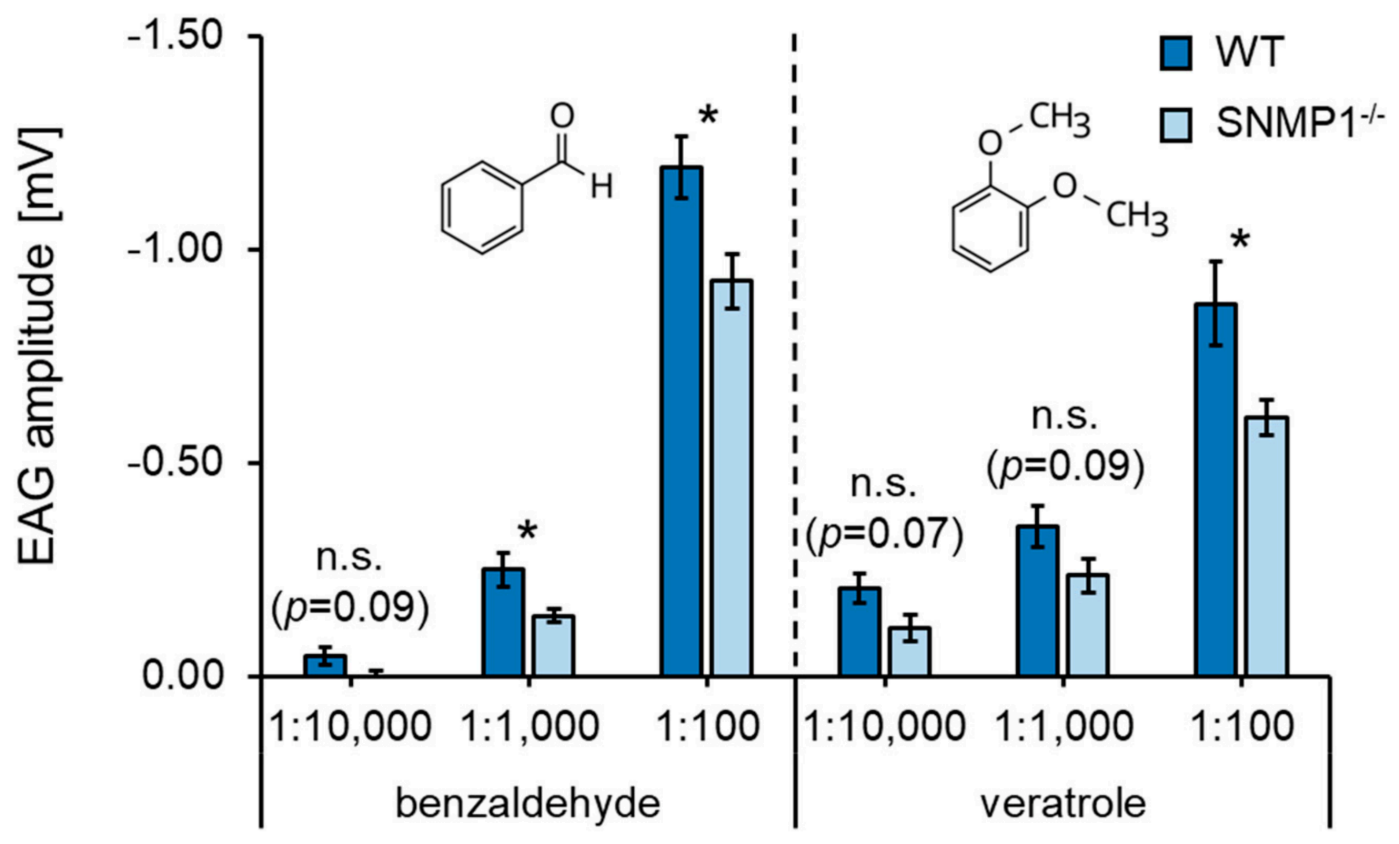
Reduction in antennal reactivity to aromatic pheromones occurs at various concentrations in SNMP1 mutants. EAG amplitudes evoked by benzaldehyde or veratrole at the different dilutions indicated were recorded from the right antenna of WT (*n* = 9–11) and SNMP1^−/−^ (*n* = 9–11) males (the data for the 1:100 dilutions were taken from Figure 1 for both compounds). Error bars denote SEM. Unpaired *t*-tests were used to calculate two-tailed *p*-values (*, 0.01 ≤ *p* < 0.05; n.s., no statistical significance).

In our previous EAG experiments, it has been found that reduction in antennal responses to PAN in mutants compared to WT animals was highest at the lowest PAN concentrations used, indicating that SNMP1 is particularly crucial for the sensitivity of PAN detection at low doses [26]. To scrutinize whether this also applies to other aromatic pheromones, benzaldehyde and veratrole were tested at dilutions of 1:1000 and 1:10,000 (Figure 2). These approaches revealed dose-dependent EAG responses. Accordingly, higher concentrations of benzaldehyde and veratrole evoked stronger responses in the antennae of both genotypes. However, for all concentrations tested, the responsiveness to benzaldehyde and veratrole was clearly lower in the antennae of SNMP1 mutants (Figure 2). For benzaldehyde, at dilutions of 1:100 and 1:1000, antennal responsiveness was significantly reduced in SNMP1^−/−^ animals, whereas at a dilution of 1:10,000, no statistically significant differences between WT and SNMP1^−/−^ animals were found (Figure 2). However, for this dilution, the antennal responsiveness to benzaldehyde was virtually abolished in SNMP1^−/−^ locusts. Thus, the reduction in the benzaldehyde-induced reactivity in mutant versus WT animals was clearly higher at a dilution of 1:10,000 than at dilutions of 1:1000 (~43% decrease) or 1:100 (~22% decline). Likewise, for veratrole, the gap between the antennal response of WT and SNMP1^−/−^ males was higher for a dilution of 1:10,000 (~45% decrease in mutants) than at dilutions of 1:1000 (33% reduction in mutants) or 1:100 (30% decline), respectively. Collectively, these findings suggest that SNMP1 could be especially crucial for the detection of lower concentrations of aromatic pheromone compounds, such as benzaldehyde and veratrole.

### 2.2. EAG Responses to Certain Non-Aromatic Pheromone Compounds Are Affected in SNMP1^−/−^ Animals

**Figure 3:**
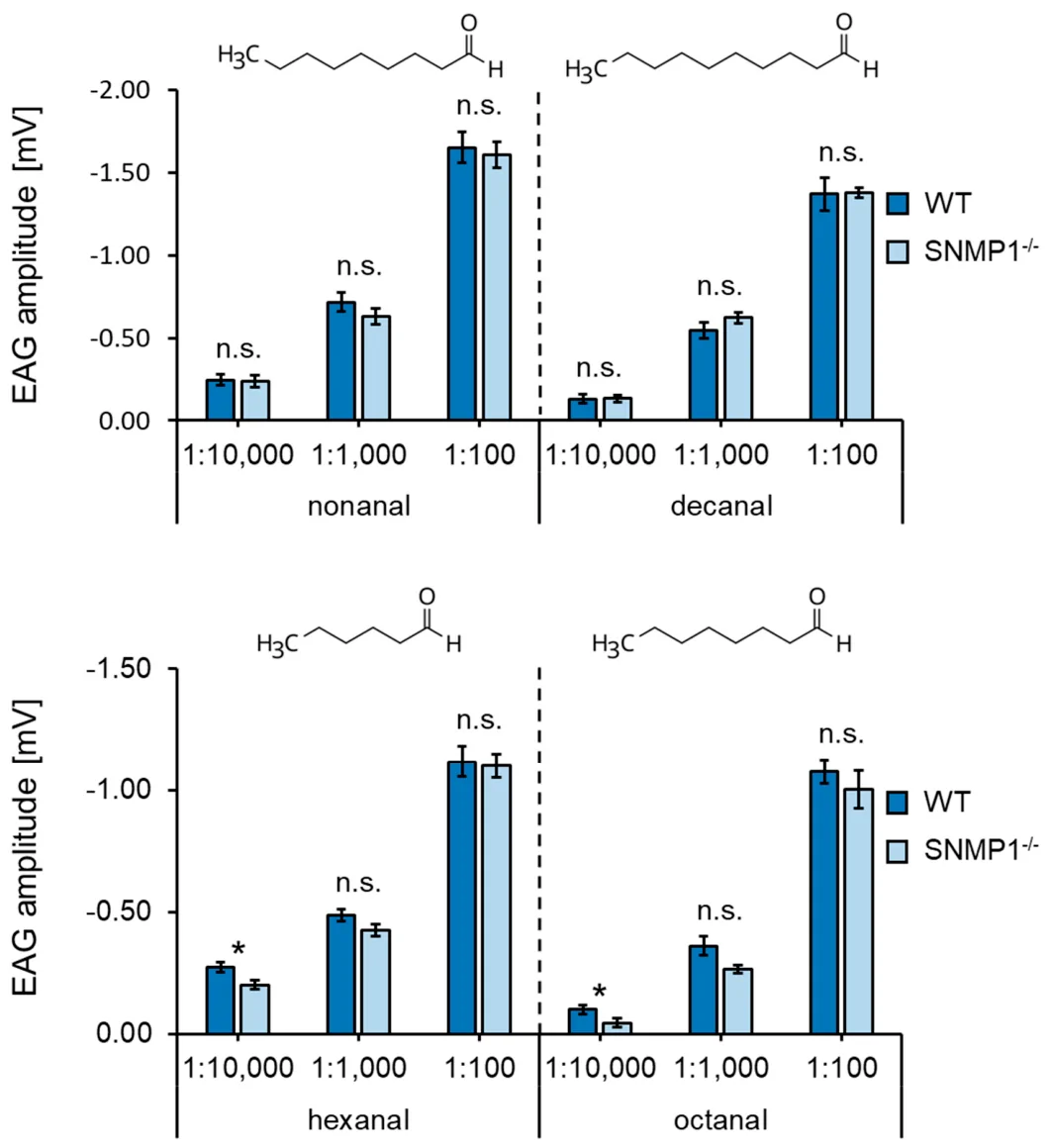
SNMP1 significantly contributes to the detection of acyclic and aliphatic desert locust pheromones with shorter carbon chains. The **upper** panel displays mean EAG responses to different dilutions of nonanal and decanal in WT (*n* = 10–13) and SNMP1^−/−^ (*n* = 11–12) males, while the **lower** panel shows EAG amplitudes to different dilutions of hexanal and octanal of WT (*n* = 11–13) and SNMP1^−/−^ (*n* = 9–12) males. Error bars indicate SEM and two-tailed *p*-values were determined by unpaired *t*-tests (*, 0.01 ≤ *p* < 0.05; n.s., no statistical significance).

In flies and moths, SNMP1 is essential for receiving long-chain, non-aromatic (aliphatic) pheromones, such as cVA or (Z)-11-hexadecenal [10,17,18]. Interestingly, some aliphatic pheromonal compounds have also been reported for the desert locust, including hexanal, octanal, nonanal, and decanal, which are part of an aggregation pheromone blend of the nymphs [32]. Importantly, these chemicals comprise clearly shorter carbon chains compared to the aliphatic pheromones in flies and moths, like cVA and (Z)-11-hexadecenal. To investigate the role of SNMP1 for detecting aliphatic pheromone compounds in *S. gregaria*, EAG experiments were performed, applying the above-mentioned aldehydes with carbon chains ranging from C6 (hexanal) to C10 (decanal) in dilutions of 1:100, 1:1000, and 1:10,000. Comparing EAG amplitudes between WT and SNMP1^−/−^ animals, no statistically significant difference could be observed for any of the tested dilutions of nonanal and decanal, respectively (upper panel in Figure 3). Similar results were obtained when hexanal or octanal were used in dilutions of 1:100 or 1:1000 (lower panel in Figure 3). However, at a dilution of 1:10,000, EAG amplitudes in mutants were lower than in WT animals, with statistically significant differences observed for both substances (lower panel in Figure 3). Consequently, it can be inferred that SNMP1 might be involved in the detection of aliphatic aldehydes with eight or fewer C-atoms, which serve as pheromonal compounds, especially when these chemicals are present in low concentrations. In contrast, aliphatic aldehyde pheromone compounds with longer chains, like nonanal or decanal, appear to be detected independently of SNMP1.

### 2.3. Responsiveness to Benzenoid Plant Volatiles Is Decreased in the Antenna of SNMP1^−/−^ Desert Locusts

**Figure 4:**
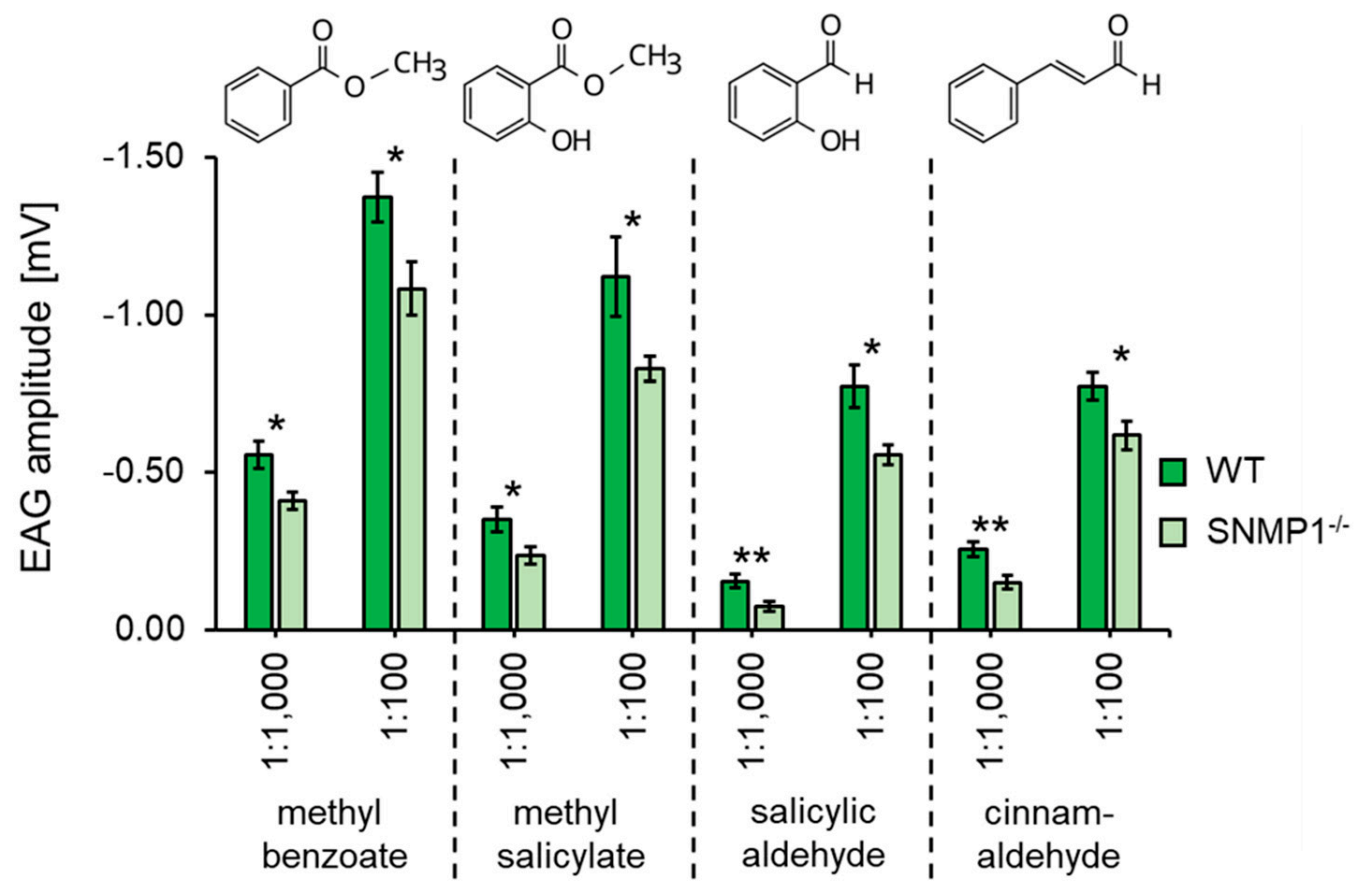
SNMP1 contributes to the antennal reception of given benzenoid plant volatiles. EAG reactivity to a set of benzenoid plant volatiles recorded from the right antenna of WT (*n* = 9–12) and SNMP1^−/−^ (*n* = 10–12) males is given. The tested dilutions are indicated. Error bars denote SEM. Unpaired *t*-tests were employed to calculate two-tailed *p*-values (**, 0.001 ≤ *p* < 0.01; *, 0.01 ≤ *p* < 0.05).

In herbivorous desert locusts, SNMP1 seems to play a crucial role in detecting benzenoid pheromone compounds and semiochemicals, such as acetophenone, benzaldehyde, veratrole, 4-vinylveratrole, and guaiacol (Figure 1). Interestingly, these substances are also found in (a number of) plants [33,34,35,36], prompting us to speculate that SNMP1 might be involved in the detection of other benzenoid substances from plants. Importantly, benzenoids constitute the second largest class of (plant) VOCs. They significantly contribute to scents as well as fruit flavors in many plant species and serve important functions in plants, including protection against herbivores [52,53,54]. As an initial step to analyze the relevance of SNMP1 for detecting aromatic plant compounds having a benzene/phenyl ring similar to the pheromonal substances acetophenone, benzaldehyde, veratrole, and 4-vinylveratrole, we applied the plant-derived benzenoids methyl benzoate, methyl salicylate, salicylic aldehyde, and cinnamaldehyde (trans-3-phenyl-2-propenal) [55,56,57] in EAG recordings of male WT and SNMP1^−/−^ locusts. Exposure to the above-described plant benzenoids in dilutions of 1:100 and 1:1000 revealed significantly lower mean EAG amplitudes in SNMP1^−/−^ locusts than in WT animals (Figure 4), indicating the involvement of SNMP1 in the detection of these aromatic plant volatiles. Moreover, this difference in the strength of EAG responses between WT and mutant animals increased when exposed to the 1:1000 dilution compared to the 1:100 dilution for methyl benzoate (from ~21% to ~26%), methyl salicylate (from ~26% to ~33%), salicylic aldehyde (from ~28% to 52%), and cinnamaldehyde (from ~20% to ~41%). Taken together, these findings suggest that SNMP1 is not only relevant for the detection of aromatic plant volatiles but also appears to enhance the sensitivity of antennal responsiveness to these substances, notably at lower concentrations.

### 2.4. Antennal Responses to Certain Cyclic Aliphatic Compounds Are Impaired in SNMP1 Mutants

**Figure 5:**
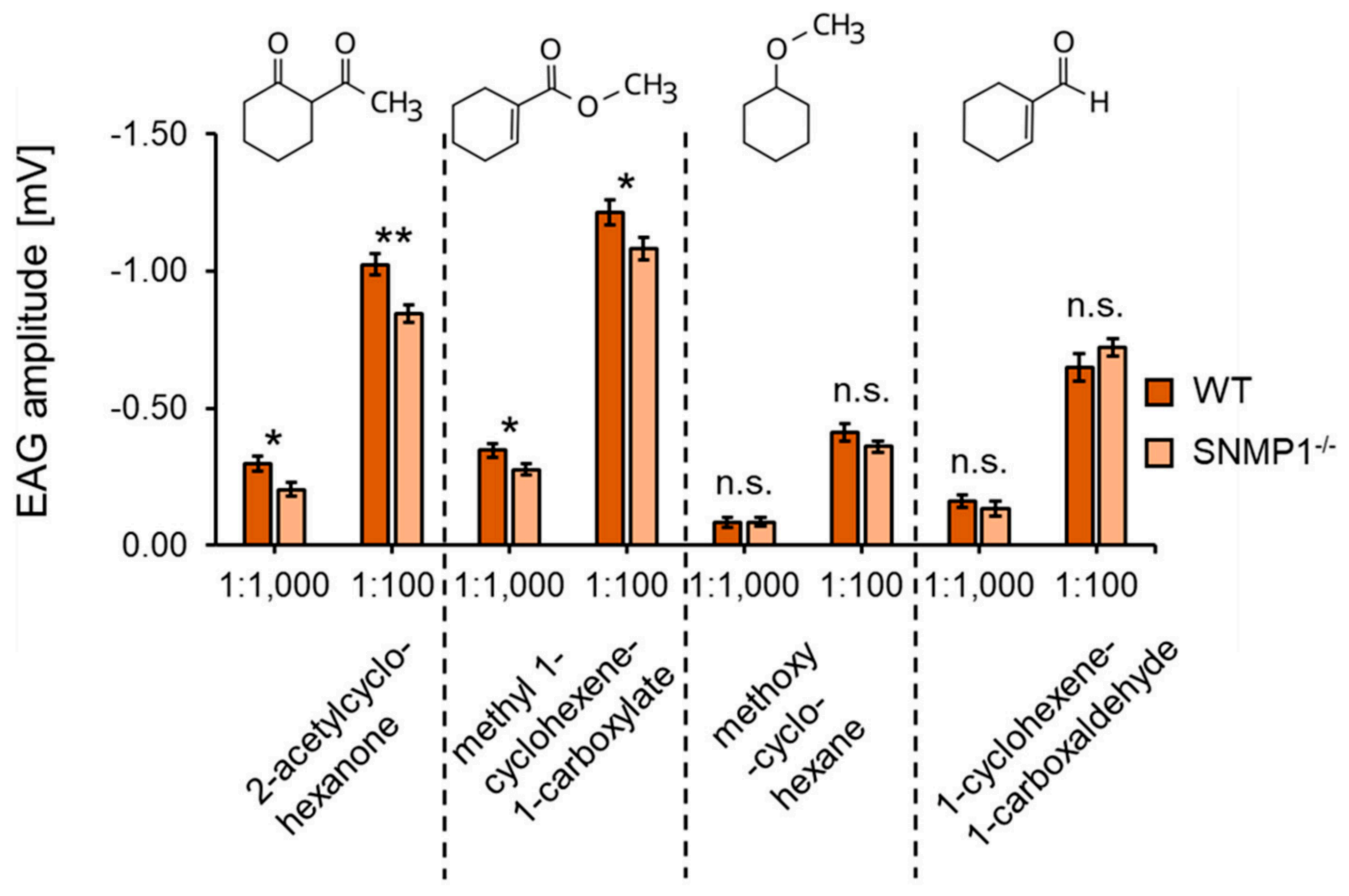
SNMP1 is involved in the detection of certain non-aromatic and cyclic substances with cyclohexyl or cyclohexenyl groups. Mean EAG responses of WT (*n* = 12–26) and SNMP1^−/−^ (*n* = 11–25) males to a set of cyclohexane and cyclohexene derivatives are depicted. Error bars indicate SEM. Two-tailed *p*-values were determined based on unpaired *t*-tests (**, 0.001 ≤ *p* < 0.01; *, 0.01 ≤ *p* < 0.05; n.s., no statistical significance).

The findings of the present study strongly indicate that SNMP1 plays a critical role for the antennal detection of aromatic substances, especially benzenoids (Figure 1 and Figure 4 as well as Figure S1). Benzenoids are endowed with a phenyl group composed of six carbon atoms joined in a hexagonal ring with conjugated double bonds. With respect to the role of SNMP1 in receiving benzenoids, it is unclear which part of these molecules is relevant for the involvement of SNMP1 in the detection of such compounds. It might be the aromatic phenyl group. Alternatively, a hexagonal ring with six carbon atoms lacking conjugated double bonds, such as a cyclohexyl or a cyclohexenyl group, could already be sufficient for the involvement of SNMP1. Although, at first glance, the aromatic phenyl group resembles aliphatic cyclohexyl or cyclohexenyl groups, there are considerable structural differences between them. Notably, while the phenyl group is planar, the cyclohexyl and cyclohexenyl groups are non-planar (chair conformation for the cyclohexyl group and half-chair conformation for the cyclohexenyl group). Therefore, another series of experiments was conducted to determine whether SNMP1 could also contribute to the antennal reception of non-aromatic compounds with an aliphatic six-carbon ring (cyclohexyl or cyclohexenyl group). To minimize possible effects of differing side chains, we opted for chemicals with identical or at least similar side chains to some of the above-mentioned benzenoids, which require SNMP1 for proper detection. Consequently, in EAG recordings, the ketone 2-acetylcyclohexanone (similar side chain to acetophenone), the ester methyl 1-cyclohexene-1-carboxylate (identical side chain to methyl benzoate), the ether methoxycyclohexane (identical side chain to anisole), and the aldehyde 1-cyclohexene-1-carboxaldehyde (identical side chain to benzaldehyde) were tested in dilutions of 1:100 and 1:1000. While for the two latter substances, no statistically significant differences were found between WT and mutant animals, in the case of 2-acetylcyclohexanone and methyl 1-cyclohexene-1-carboxylate, antennal responses in mutants were significantly reduced at both concentrations (Figure 5). As for benzenoids (Figure 2 and Figure 4) and acyclic aldehyde pheromone compounds (Figure 3), the differences in the amplitude of EAG responses between WT and SNMP1^−/−^ males increased when exposed to the 1:1000 dilution compared to the 1:100 dilution in the case of 2-acetylcyclohexanone (from ~18% to ~31%) and methyl 1-cyclohexen-1-carboxylate (from ~11% to ~20%) (Figure 5). Thus, in summary, the involvement of SNMP1 in the recognition of cyclic substances with a hexagonal ring is not limited to aromatic benzenoids, but apparently also applies to some aliphatic cyclic substances with a cyclohexyl or cyclohexenyl group.

### 2.5. Antennal Responsiveness to Acyclic Aliphatic Plant Compounds Is Altered in SNMP1 Mutants

**Figure 6:**
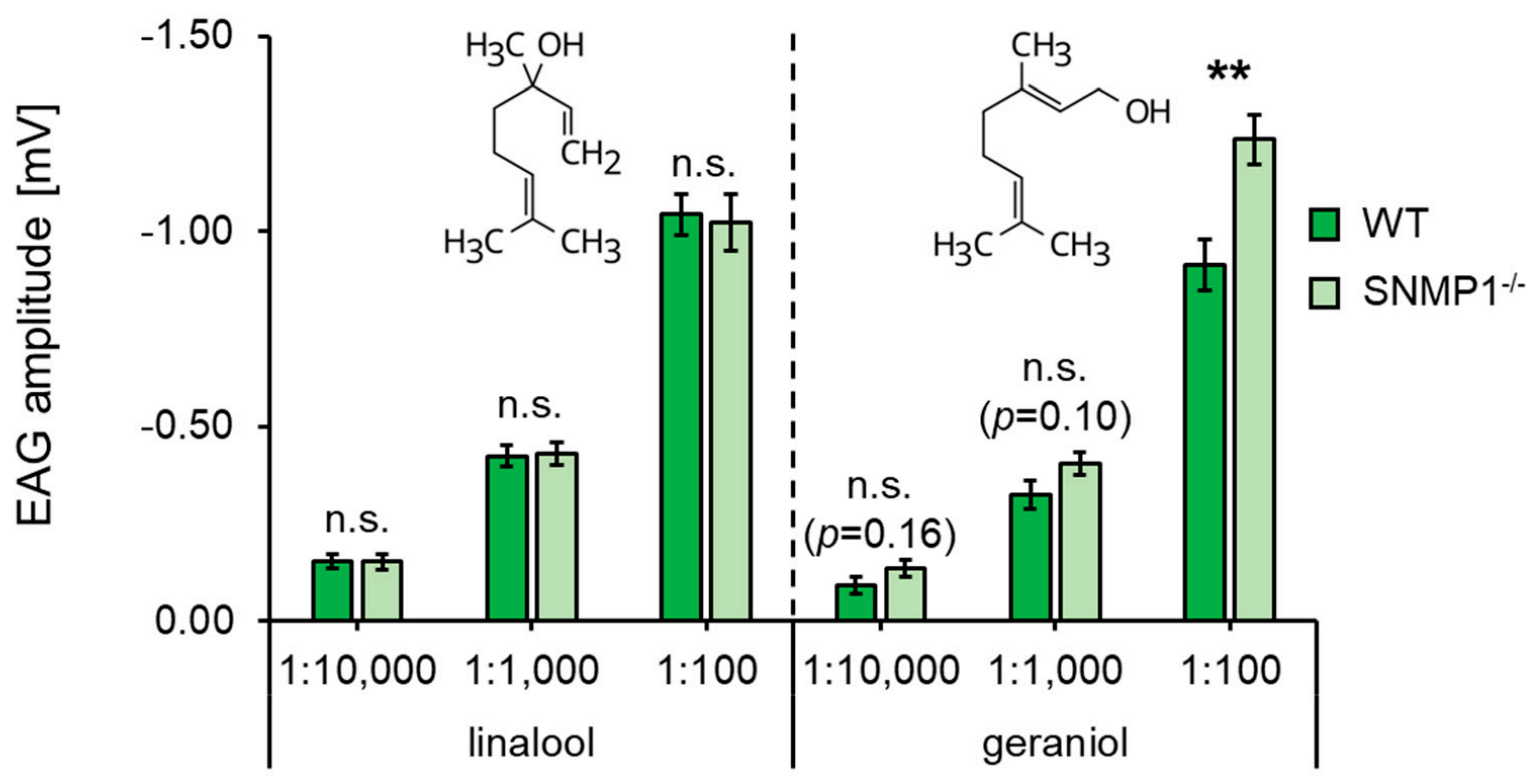
Role of SNMP1 for the antennal reception of the plant terpenoids linalool and geraniol. Antennal responsiveness to the denoted dilutions of the tertiary alcohol linalool and the primary alcohol geraniol in WT (*n* = 11–13) and SNMP1^−/−^ (*n* = 10–13) males are given. Error bars indicate SEM and two-tailed *p*-values were calculated by unpaired *t*-tests (**, 0.001 ≤ *p* < 0.01; n.s., no statistical significance).

**Figure 7:**
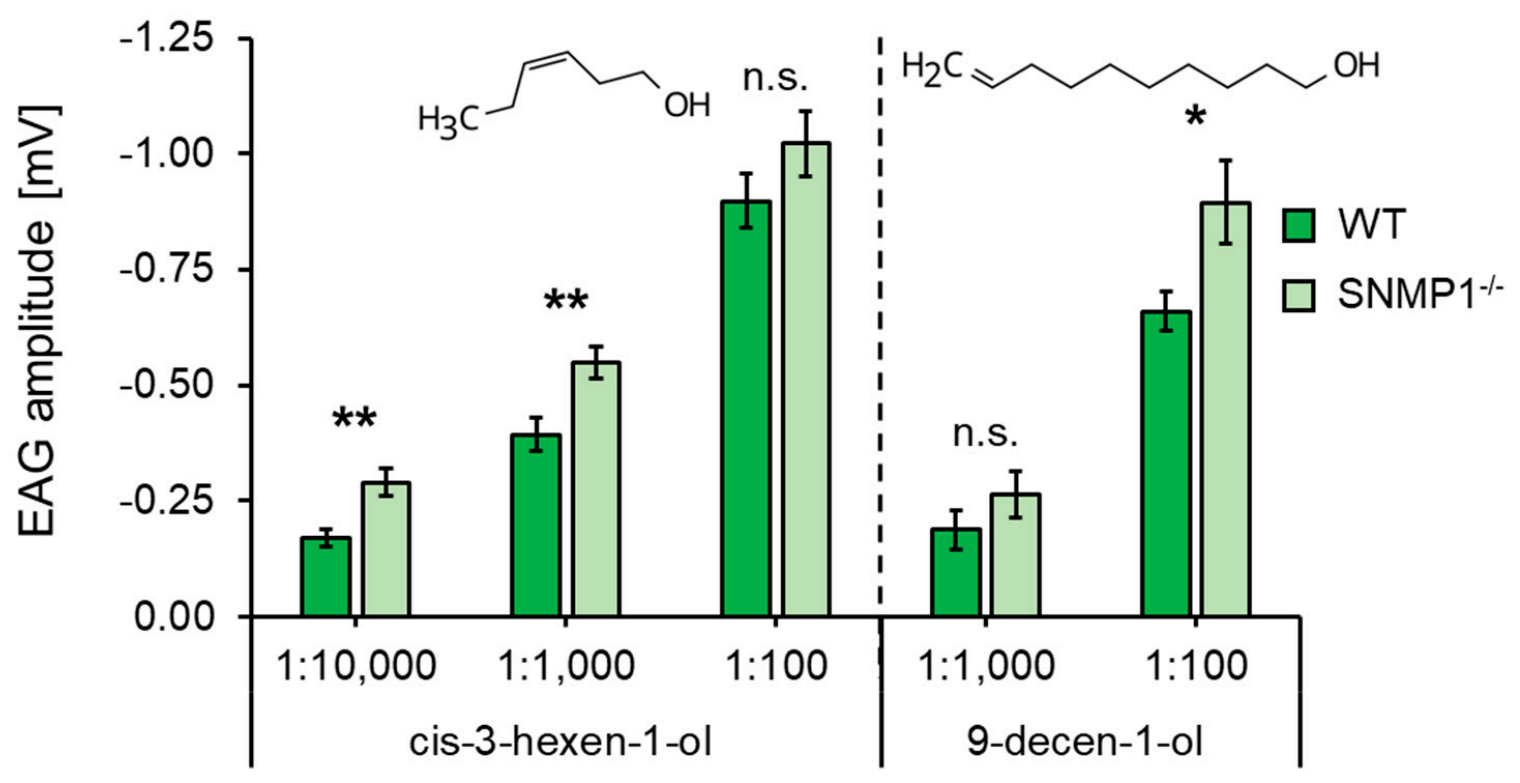
Antennal reactivity to primary alcohols from plants is increased in SNMP1 mutants. EAG amplitudes from the right antenna of WT (*n* = 11–13) and SNMP1^−/−^ (*n* = 12–13) males were recorded upon exposure to different dilutions of the primary alcohols cis-3-hexen-1-ol or 9-decen-1-ol, respectively. The SEM is denoted by error bars and two-tailed *p*-values were determined by unpaired *t*-tests (**, 0.001 ≤ *p* < 0.01; *, 0.01 ≤ *p* < 0.05; n.s., no statistical significance).

**Figure 8:**
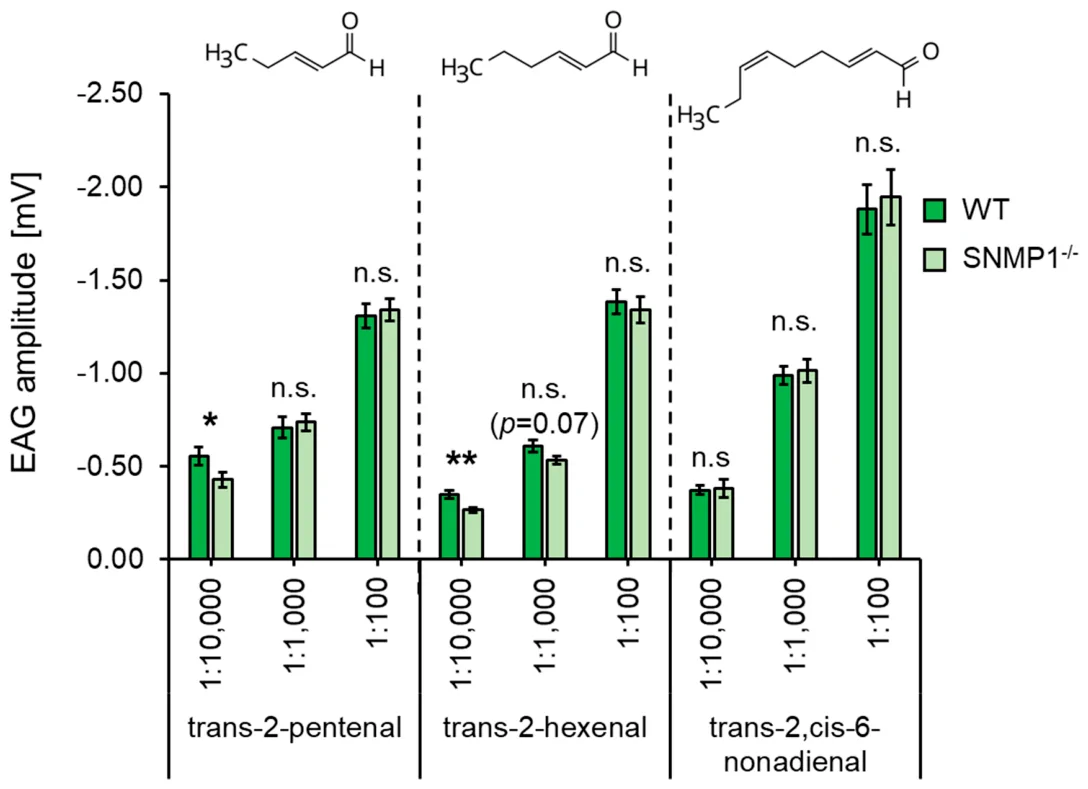
Role of SNMP1 for the detection of fatty acid-derived plant aldehydes. Mean EAG responses of WT (*n* = 9–15) and SNMP1^−/−^ (*n* = 7–15) males induced by different dilutions of trans-2-pentenal, trans-2-hexenal, or trans-2,cis-6-nonadienal are shown. Error bars indicate SEM and two-tailed *p*-values were calculated based on unpaired *t*-tests (**, 0.001 ≤ *p* < 0.01; *, 0.01 ≤ *p* < 0.05; n.s., no statistical significance).

The observation that SNMP1 facilitates proper detection of aromatic plant odorants prompted us to scrutinize whether SNMP1 is also involved in the antennal reception of other classes of VOCs in *S. gregaria*, notably acyclic and aliphatic plant volatiles. Interestingly, in this regard, in a given subpopulation of OSNs in *Drosophila melanogaster*, SNMP1 was found to be critical for a proper response to the acyclic and aliphatic alcohol farnesol [44], an odorant of citrus fruits that apparently does not serve as a pheromone in vinegar flies [45,46,47]. Farnesol belongs to the VOC group of terpenoids [58] which constitute the largest (and most diverse) class of VOCs [40] and exhibit a chemical structure that differs greatly from that of benzenoids. To scrutinize if SNMP1 could also contribute to the detection of terpenoids in the desert locust, EAG recordings were performed with farnesol. Yet, stimulation of both WT and SNMP1^−/−^ antennae with farnesol (diluted 1:100) elicited only very weak EAG responses (data not shown), which did not allow for further data analysis. Therefore, two other plant-derived terpenoids, namely linalool and its isomer geraniol [59,60], were utilized in dilutions of 1:100, 1:1000 and 1:10,000 for antennal recordings. Consistent with a recent study [26], exposing the antennae of male WT and SNMP1^−/−^ desert locusts to linalool (3,7-dimethyl-1,6-octadien-3-ol), EAG responses of the WT individuals did not differ significantly from those of the mutants at all dilutions tested (Figure 6). Unexpectedly, however, the mutants exhibited an increased response to geraniol (trans-3,7-dimethyl-2,6-octadien-1-ol), another acyclic monoterpene alcohol (Figure 6). This result provides first evidence that SNMP1 might be involved in the detection of some terpenoid plant volatiles. With regard to the structural formula, it should be noted that linalool is a tertiary alcohol, while geraniol is a primary alcohol. Consequently, we tested two additional primary and acyclic alcohols from plants in EAG experiments, cis-3-hexen-1-ol and 9-decen-1-ol [61,62]. Similar to geraniol, application of these substances induced EAG amplitudes that were considerably increased in SNMP1 mutants compared to WT conspecifics (Figure 7). Noteworthy, the difference in the EAG amplitudes between WT and SNMP1-deficient animals was highest for all three primary alcohols tested when the strongest dilution (1:10,000 for geraniol and cis-3-hexen-1-ol or 1:1000 in the case of 9-decen-1-ol) was used (geraniol: 46% increase in mutants for 1:10,000 dilution versus 35% for 1:100 dilution; cis-3-hexen-1-ol: 70% rise in SNMP1^−/−^ locusts for 1:10,000 dilution versus 14% for 1:100 dilution; 9-decen-1-ol: 40% upsurge in SNMP1^−/−^ locusts for 1:1000 dilution versus 35% for 1:100 dilution; Figure 6 and Figure 7). Thus, taken together, these findings demonstrate that the presence of SNMP1 has an impact on the antennal reception of primary alcohols. Intriguingly, unlike its role in detecting other chemicals, the absence of SNMP1 enhances responsiveness to primary alcohols. It is also noteworthy that geraniol, cis-3-hexen-1-ol, and 9-decen-1-ol, although they are primary alcohols, belong to different classes of VOCs: While geraniol is a terpenoid, cis-3-hexen-1-ol and 9-decen-1-ol are fatty acid derivatives belonging to another important group of plant volatiles, which includes various alcohols, aldehydes, and esters [40,41]. For plant aldehydes arising from fatty acids, it is important to note that in the present study, we found that SNMP1 is involved in the detection of some acyclic and aliphatic aldehydes with relatively short carbon chains, including hexanal and octanal (Figure 3), which are part of an aggregation pheromone blend of *S. gregaria* nymphs, but also occur in plants [32,38,63,64]. Therefore, to assess the relevance of SNMP1 for detecting other plant-derived aliphatic aldehydes, such as trans-2-pentenal, trans-2-hexenal (trans-2-hexen-1-al), and trans-2,cis-6-nonadienal [65,66,67], EAG recordings were conducted in which these chemicals were applied to antennae of WT and SNMP1^−/−^ males in dilutions of 1:100, 1:1000, and 1:10,000. In the case of trans-2,cis-6-nonadienal, the compound with the longest carbon chain, no significant differences between WT and mutant animals were detectable at any of the dilutions tested (Figure 8). Likewise, antennal reactivity for trans-2-pentenal and trans-2-hexenal did not differ significantly between these genotypes when these compounds were applied in dilutions of 1:100 and 1:1000 (Figure 8). In contrast, at a dilution of 1:10,000, EAG responses were significantly reduced in mutants for both substances in comparison to WT conspecifics (by ~23% for trans-2-pentenal and by ~24% for trans-2-hexenal; Figure 8), corroborating the view that SNMP1 contributes to the detection of such acyclic plant aldehydes with a rather short carbon chain; at least at lower concentrations. With respect to other VOCs arising from fatty acids, the role of SNMP1 for detecting the plant-derived ester cis-3-hexenyl acetate [38] was investigated in EAG experiments in which this substance was employed in dilutions of 1:100, 1:1000, and 1:10,000. In these approaches, no significant differences in the antennal reactivity were detectable between WT and mutant animals in any dilution tested (Figure S2), indicating that SNMP1 does not play a role for receiving this ester.

### 2.6. SNMP1 Plays a Crucial Role in Attracting Desert Locusts to Food Plant Scents

**Figure 9:**
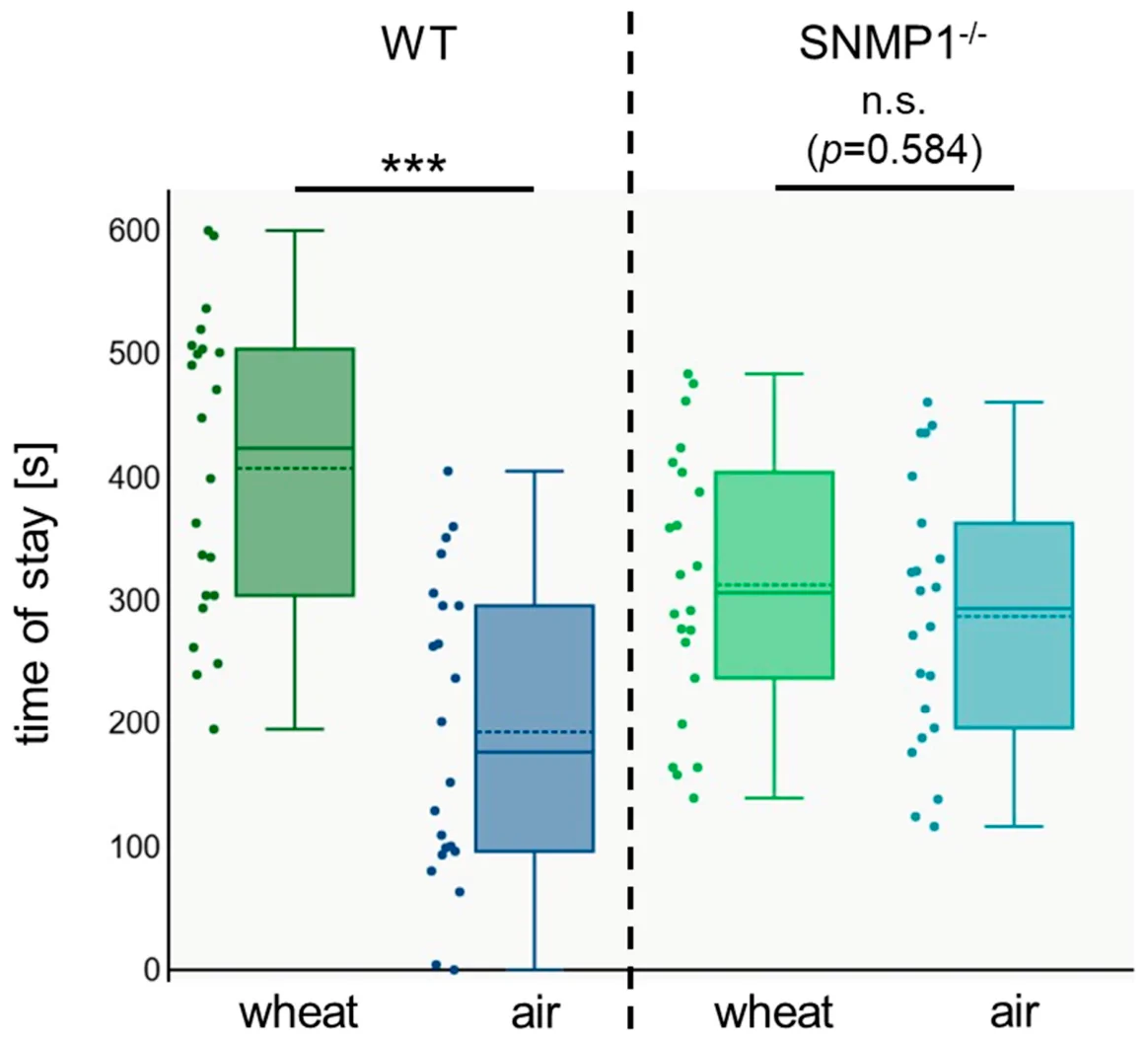
Attraction to food plant scents is reduced in SNMP1 mutants compared to WT conspecifics. The duration of the stay in the half of the arena suffused with the scent of wheatgrass and the other half of the arena ventilated with air was measured for WT (*n* = 22) and SNMP1^−/−^ (*n* = 22) desert locusts during a 600 s period. The results are depicted as a box plot. The height of the boxes represents the interquartile range. In the boxes, the dashed line denotes the mean, whereas the solid line indicates the median. The whiskers extend beyond the interquartile range and reflect the spread of the data, while the points to the left of the boxes show the actual measured values. The box plot was created using the “Advanced Boxplot Maker” website, provided by “Statistics Kingdom” (https://www.statskingdom.com/advanced-boxplot-maker.html, accessed on 30 August 2026). Paired *t*-tests were conducted to determine two-tailed *p*-values (***, *p* < 0.001; n.s., no statistical significance).

The results of the EAG experiments indicate that SNMP1 is critical for the detection of given plant-derived odorants in *S. gregaria* (Figure 4 and Figure 8), leading to the question of whether SNMP1 is also essential for attracting desert locusts via plant scents to food plants. To address this issue, starved WT and SNMP1^−/−^ animals were subjected to dual-choice olfactometer tests in an arena. In these experiments, the scent of wheatgrass (food plant) was applied to one side of the olfactometer, while pure air (control) was delivered to the other side (Figure S3). Investigating a period of 600 s, a statistically significant difference was found for WT animals regarding the duration spent in the half of the arena suffused with the scent of wheatgrass versus the other half of the arena that was ventilated with air, as the locusts stayed more than twice as much time in the wheatgrass-scented half as in the other half. In fact, WT animals spent ~68% of the observation period in the half with the wheatgrass odor and only ~32% of the time in the other half (Figure 9). By contrast, analyzing the behavior of SNMP1-deficient desert locusts revealed no significant statistical preference in the duration of their stay since they spent ~52% of the time in the half with the wheatgrass scent and ~48% in the other half (Figure 9). Thus, SNMP1 seems to be critical for attracting desert locusts to food plant odors.

### 2.7. Expression of ORs Co-Expressed with SNMP1 in WT Desert Locusts Is Not Impaired in the Antenna of SNMP1 Mutants

**Figure 10:**
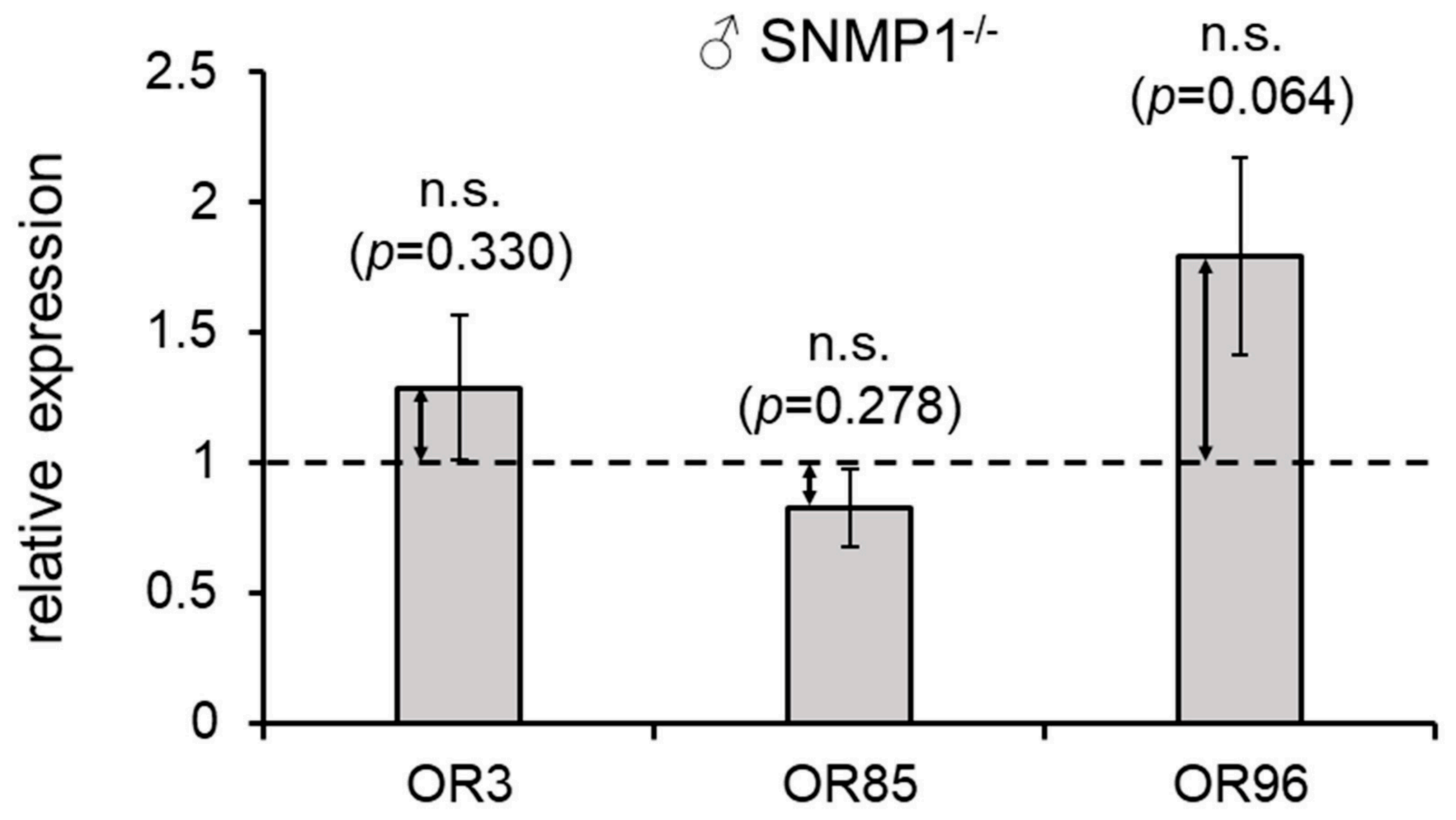
The expression of OR types co-expressed with SNMP1 in WT locusts is not significantly reduced in the antenna of SNMP1^−/−^ males. Quantitative PCR approaches were conducted with antennal cDNA from WT and SNMP1^−/−^ males as well as specific primer pairs for OR3, OR85, and OR96. For normalization, GAPDH was used as an endogenous standard. The bars represent the mean relative expression of OR3, OR85, and OR96 in the antenna of SNMP1^−/−^ males. The broken horizontal line denotes a relative expression of 1, i.e., an expression level of the respective OR type corresponding to that in the antenna of WT males. Double arrows mark the mean difference between the expression rate of the relevant ORs in WT and mutant animals. The results are derived from six (OR3 and OR96) and five (OR85) independent experiments, respectively. Error bars indicate the SEM. Two-tailed *p*-values were determined by unpaired *t*-tests (n.s., no statistical significance).

According to a current model of SNMP1 function in *Drosophila* flies, SNMP1 acts as a co-receptor that funnels ligands via its tunnel-like ectodomain to a nearby heteromeric olfactory receptor formed by a ligand-binding OR subunit and the odorant receptor co-receptor ORCO [20]. Consequently, in the absence of SNMP1, the access of pheromonal and odorant molecules to ligand-binding OR proteins might be impeded. Alternatively, reduced olfactory responses of SNMP1^−/−^ locusts to given chemicals could be due to indirect effects, e.g., elimination of functional SNMP1 expression might also impair the expression of OR types that are co-expressed with SNMP1 in WT locusts. In this context, it is worth noting that, according to recent reports, knocking out ORCO in honeybees and *Drosophila* flies leads to a substantially diminished antennal expression of numerous OR types in the antenna [68,69]. Against this background, we scrutinized a potentially reduced OR expression in SNMP1^−/−^ locusts by quantitative PCR (qPCR) experiments with OR-specific primer pairs and antennal cDNA from WT and SNMP1^−/−^ males. Since the OR types for substances with decreased EAG responses in SNMP1 mutants are unknown, we exemplarily analyzed expression of three OR types (OR3, OR85, and OR96) reported to be co-expressed with SNMP1 in antennal OSNs of the desert locust [24,25]. While OR3 is one of the few OR types expressed in OSNs of trichoid sensilla, OR85 and OR96 are found in neurons of basiconic sensilla [70]. The qPCR approaches for OR85 disclosed a non-significant reduction in the antennal mRNA expression level in SNMP1-deficient animals (Figure 10). By contrast, the expression of mRNA encoding OR3 or OR96 appeared to be higher in mutants (Figure 10). For both ORs, these increases in the mRNA level were statistically not significant. In summary, our results indicate that the observed impairment of electrical responsiveness to certain compounds in the antenna of SNMP1^−/−^ locusts is not due to compromised expression of OR types that are co-expressed with SNMP1 in OSNs of WT animals.

## 3. Discussion

In insects, SNMP1 is considered an important signaling element of antennal OSNs for receiving pheromonal cues. In fact, earlier studies provided evidence that SNMP1 is highly conserved across insects and critical for the detection of aliphatic pheromonal compounds with longer carbon chains in holometabolous moth and fly species [10,17,18,19]. However, such pheromone substances have not been reported for the hemimetabolous desert locust *S. gregaria*; by contrast, most of the pheromones described so far for this species are (aromatic) benzenoids with rather short side chains, including acetophenone, benzaldehyde, veratrole, and 4-vinylveratrole [29,30,31]. Our experiments revealed that the EAG responses for these substances were significantly lower in SNMP1^−/−^ animals compared to WT conspecifics (Figure 1 and Figure 2), strongly suggesting that SNMP1 is involved in the reception of these benzenoid pheromone compounds in the antenna of *S. gregaria*, a finding that is consistent with the recent observation that SNMP1 contributes to the detection of the aromatic pheromone phenylacetonitrile (PAN) in OSNs of the desert locust [26]. Consequently, SNMP1 appears to play a general role in the reception of aromatic pheromones in *S. gregaria*, indicating that SNMP1 does not seem to be only of crucial relevance for detecting aliphatic pheromones. Interestingly, aromatic pheromone substances are not confined to locusts; for instance, the benzenoid substance 4-methylanisole functions as an aggregation pheromone in the beetle *Protaetia brevitarsis* [71]. Thus, SNMP1 might also be involved in the detection of aromatic pheromones in other insects.

In the course of the present study, we found that SNMP1 is also crucial for detecting given aliphatic pheromone substances in desert locusts, notably hexanal and octanal (Figure 3). This observation is in line with previous reports demonstrating the involvement of SNMP1 in receiving aliphatic pheromone substances in holometabolous flies and moths [10,17,18,19]. However, the aliphatic pheromone compounds requiring SNMP1 for proper olfactory detection, including cis-vaccenyl acetate (cVA) in *Drosophila* flies [10,17] or (Z)-11-hexadecenal and (Z)-9-hexadecenal in moths [18,19], comprise rather long carbon chains. Detailed analyses in the moth *Helicoverpa armigera* have demonstrated that SNMP1 is essential in antennal OSNs for the detection of the pheromone substances (Z)-11-hexadecenal and (Z)-9-hexadecenal, each of which comprises 16 carbon atoms, whereas it is not required for receiving a shorter pheromonal compound with a similar structure, (Z)-9-tetradecenal, which has only 14 carbon atoms. Therefore, it has been proposed that SNMP1 plays an important role in detecting long-chain pheromone substances, but is not crucial for receiving pheromone compounds with shorter carbon chains [18]. Intriguingly, this scenario seems to be reversed in desert locusts. In fact, when (aliphatic) aldehydes with shorter carbon chains, i.e., hexanal and octanal, were used as stimuli, antennal reactivity was significantly lower in SNMP1 mutants compared to WT conspecifics (lower panel in Figure 3). By contrast, in case of aldehydes with an increased length of the carbon chain (nonanal and decanal), no significant differences in the EAG responses between WT and SNMP1-deficient locusts were detectable (upper panel in Figure 3). Consequently, a longer carbon chain does not seem to be a general criterion in all insects with respect to the relevance of SNMP1 for the detection of (aliphatic) pheromone compounds. This concept is consistent with recent findings demonstrating that in the beetle *Rhynchophorus ferrugineus*, SNMP1 is critical for receiving the aliphatic pheromone compounds 4-methyl-5-nonanol (ferrugineol) and 4-methyl-5-nonanone (ferrugineone) [72], whose carbon chain lengths are also clearly shorter than those of (Z)-11-hexadecenal and (Z)-9-hexadecenal.

Several of the aromatic and aliphatic pheromone substances in *S. gregaria* have also been found in (some) plants [33,34,35,36,38,63,64]. Therefore, we tested whether SNMP1 also plays a role in the antennal detection of plant-derived compounds, most notably VOCs, that do not function as pheromones in desert locusts. These approaches revealed that antennal responsiveness to a number of VOCs tested, including benzenoids (e.g., methyl benzoate and methyl salicylate) and fatty acid-derived substances (trans-2-pentenal and trans-2-hexenal), was significantly reduced in SNMP1-deficient locusts (Figure 4 and Figure 8), demonstrating that SNMP1 is critical for receiving given plant odorants. These observations are in marked contrast to previous findings in the moth *Helicoverpa armigera*, in which SNMP1 was not required for the detection of nine plant odorants tested, such as methyl benzoate, methyl salicylate, and geraniol [18]. Thus, the relevance of SNMP1 for olfactory reception might be species-specific. While in some species (e.g., moths), a functional role of this protein could be confined to pheromone detection, in other insects (e.g., locusts), SNMP1 apparently contributes to the reception of a much broader repertoire of chemicals, including VOCs. However, it has to be pointed out that in *Helicoverpa armigera*, only a small set of substances has been tested with respect to a potentially SNMP1-dependent olfactory detection of plant odorants [18]. Thus, it cannot be excluded that SNMP1 might be involved in the detection of chemicals other than pheromones in moths as well. The latter notion is also consistent with the observation that in some moth species (*Antheraea polyphemus*, *Spodoptera exigua*), SNMP1 is expressed in different sensilla types, including trichoid, intermediate and basiconic sensilla [73,74], suggesting a more widespread expression of SNMP1 in moth OSNs that might extend beyond pheromone-sensitive neurons. In *Drosophila melanogaster*, earlier findings already provided preliminary evidence for the role of SNMP1 beyond pheromone detection because SNMP1 was found to be crucial for normal responsiveness of given OSNs to the terpenoid farnesol, a plant odorant present in citrus fruits [44]. Yet, it remains largely unclear to what extent SNMP1 plays a substantial role in receiving non-pheromonal compounds in holometabolous insects. In *S. gregaria*, however, the relevance of SNMP1 for detecting a larger array of substances, including non-pheromonal compounds, is in line with the co-expression of SNMP1 with a considerable number of distinct OR types. In fact, the observation that more than 30 OR types are co-expressed with SNMP1 in subpopulations of OSNs strongly suggests that SNMP1 could contribute to the detection of a broader spectrum of chemicals in desert locusts [25]. Although the cognate ligands of the *S. gregaria* OR types that are co-expressed with SNMP1 are still unknown, the results of this study indicate that they apparently comprise pheromonal and non-pheromonal substances, including aromatic (benzenoids) as well as aliphatic (notably aldehydes) substances (Figure 1, Figure 2, Figure 3, Figure 4 and Figure 8). Intriguingly, these chemicals not only include pheromones and VOCs, but also the ketone 2-acetylcyclohexanone and the carboxylic acid derivative methyl 1-cyclohexene-1-carboxylate (Figure 5), two cyclic and non-aromatic chemicals for which, to our knowledge, no natural occurrence has been described to date. Consequently, in *S. gregaria*, it appears that SNMP1 is not specifically—or even exclusively—designated to contribute to the detection of semiochemicals, such as pheromones and key (attractant or repellent) plant odorants that control critical insect behaviors, including reproduction and food preferences. Thus, the relevance of SNMP1 for the olfactory reception of a given chemical may not be related to its biological or ecological significance, but rather to the physicochemical properties of its molecules or receptors.

Current models suggest that SNMP1 might accelerate the access of ligand molecules to their cognate ORs by directing them to the binding pocket of receptors [20], which is supposed to lie within the transmembrane regions [75]. Such a role of SNMP1 in enhancing the access of ligands to the corresponding OR types could be particularly important at low concentrations of pheromones or odorants, where a sufficiently strong response of OSNs should still be achieved despite the very limited number of ligand molecules. The concept that SNMP1 is particularly crucial for providing a highly sensitive detection of chemicals at lower concentrations is in line with our findings, indicating a dose-dependent role of SNMP1 in receiving pheromones and odorants. In fact, for all tested pheromone compounds and odorants that apparently require SNMP1 for proper detection, the difference in signal strength between the antennae of WT animals and those of SNMP1-deficient conspecifics was greatest (in percentage terms) when the lowest concentration of the chemical compound was applied (Figure 2, Figure 3, Figure 4, Figure 5 and Figure 8). Importantly, even in the absence of SNMP1, EAG responses to these compounds were detectable (Figure 2, Figure 3, Figure 4, Figure 5 and Figure 8). Similar observations were made in EAG experiments and single sensillum recordings (SSR) regarding the detection of the pheromonal substance PAN in desert locusts [26]. Collectively, these findings indicate that SNMP1 is essential for the sensitive detection of given pheromones and odorants; yet, it does not seem to be required per se for the activation of OSNs by these chemicals. This result appears to contradict previous observations, according to which pheromone-induced responses in OSNs of the T1 sensilla of *Drosophila melanogaster* and in B-neurons of the type C trichoid sensilla of *Helicoverpa armigera* were (almost) completely abolished in SNMP1-mutant animals [10,17,18,20,76]. However, in *Helicoverpa armigera*, the so-called A-neurons in type A trichoid sensilla are still activated by their pheromone ligand in animals lacking SNMP1, although with diminished sensitivity, i.e., the responsiveness of these neurons to the pheromone is significantly lower in moths with an SNMP1 mutation compared to WT conspecifics [18]. Thus, in these cells, SNMP1 is crucial for the sensitivity of pheromone-induced responses, but not mandatory for the ability to react to pheromones per se. A similar scenario might account for OSNs in desert locusts. Alternatively, it is also conceivable that pheromones and odorants to which SNMP1 mutants are less responsive also stimulate certain OR types expressed in SNMP1-negative OSNs in WT *S. gregaria*. Accordingly, the observed remaining EAG responses to such compounds in SNMP1 mutants could be attributed to these latter populations of OSNs, which would retain their reactivity in individuals with SNMP1 deficiency. In any case, the reduced reactivity to certain pheromones and odorants in SNMP1 mutants raises the question of whether a decrease (or increase) in olfactory sensitivity has any substantial behavioral or physiological consequences at all in locusts. In this regard, it was recently observed for the pheromonal compound PAN that a diminished (but not abolished) antennal responsiveness to this substance in SNMP1-deficient desert locusts significantly affects the behavior of such animals, demonstrating that the SNMP1-mediated sensitivity in olfactory detection controls insect behavior [26]. In line with these earlier findings, the behavioral experiments of this study showed that the preference for food odors in desert locusts is impaired in SNMP1-deficient individuals (Figure 9). Consequently, in *S. gregaria*, SNMP1 is not only crucial for detecting plant-released substances such as certain VOCs, but also for behaviors governed by food plant scents. This notion is consistent with previous observations in other hemimetabolous insects, including the planthopper *Sogatella furcifera* and the aphid *Rhopalosiphum padi*, demonstrating that SNMP1 is critical for seeking behavior and attraction to host plants [48,49]. Collectively, these findings corroborate the concept that the role of SNMP1 in hemimetabolous insects extends beyond the detection of pheromonal compounds.

In the course of the present study, we unexpectedly found that electrical reactivity for some alcoholic odorants (geraniol, cis-3-hexen-1-ol, and 9-decen-1-ol) was enhanced in the antenna of SNMP1-deficient animals (Figure 6 and Figure 7), a phenomenon that, to our knowledge, has not yet been described. The molecular and physiological mechanisms that underlie this increased responsiveness to certain primary alcohols in the absence of functional SNMP1 remain unclear. One possibility may be that responses to given odorants in WT locusts could be suppressed by SNMP1 in certain OSNs. In this regard, a mechanism termed lateral inhibition might be considered. During lateral inhibition, electrical responsiveness of one OSN to an odorant is suppressed by odorant-induced activation of a neighboring OSN housed in the same sensillum, presumably through ephaptic coupling [77]. In a scenario, in which two adjacent OSNs (one of them expresses SNMP1 while the other is SNMP1-negative) are activated by the same odorant (e.g., a primary alcohol such as cis-3-hexen-1-ol), in WT animals, the activation of one cell would reduce the response of the other one and vice versa, causing the signals of the two cells to cancel each other out. As a consequence, the resulting signal evoked by this odorant should be weak—at least for this sensillum. In the same sensillum of an SNMP1 mutant, however, the odorant-induced response of the OSN expressing the non-functional SNMP1 would be attenuated or even absent, resulting in suppressed lateral inhibition of the SNMP1-negative OSN, thus preventing mutual signal cancellation. It is currently elusive whether in *S. gregaria*, adjacent OSNs exist in antennal sensilla expressing receptor types activated by the same odorant. It is noteworthy that cis-3-hexen-1-ol seems to elicit activation of at least seven out of forty-two ORs tested in the related locust species *Locusta migratoria* [78]; yet, it is unknown whether these cis-3-hexen-1-ol-reactive ORs are expressed in neighboring OSNs.

As an alternative to lateral inhibition, it could be taken into consideration that a rise in reactivity to given alcohols in SNMP1-mutant locusts might be due to odorant-evoked direct inhibition of OSNs. In *Drosophila* flies, certain inhibitory odorants, including the primary alcohol geraniol, have been identified to significantly reduce the constitutive activities of ORs, leading to diminished basal spike firing in OSNs [79]. Interestingly, inhibition of olfactory neurons by odorants has already been described for OSNs on the antennae of *S. gregaria* [80]. If inhibition of given ORs via a corresponding inhibitory odorant would rely on SNMP1, it is conceivable that electrical signals recorded upon application of this odorant are higher in SNMP1 mutants than in WT individuals. Such a potential SNMP1-mediated inhibition of ORs might be based on effective funneling of inhibitory odorants to ORs via SNMP1, similar to the above-mentioned model for SNMP1-mediated activation of ORs [20]. In this context, it is also interesting to note that the difference (in percentage terms) between EAG amplitudes in WT and SNMP1^−/−^ animals was highest at the lowest odorant concentrations tested for these compounds (geraniol, cis-3-hexen-1-ol, and 9-decen-1-ol; Figure 6 and Figure 7), similar to our observations for chemical cues for which responsiveness was reduced in SNMP1 mutants (such as benzaldehyde, veratrole, hexanal, and octanal; Figure 2 and Figure 3). Therefore, it is tempting to speculate that SNMP1 not only contributes to the sensitivity of OSN activation by chemical cues but also impacts on OSN inhibition. It is not yet clear whether odorant-triggered inhibition of ORs in *S. gregaria* has any effect on the animal, particularly on its behavior. For *Drosophila* flies, however, it has been reported that odorant-induced inhibition of OSNs influences several behaviors (including attraction and avoidance), in a manner similar to the stimulation of olfactory neurons by odorants [79]. Consequently, the reduced responsiveness to certain alcohols observed in WT desert locusts, which is associated with the presence of a functional SNMP1, may have behavioral (or physiological) significance.

Overall, our findings—which indicate that SNMP1 contributes to the sensitive detection of an obviously broader range of different pheromones and odorants in locusts—strongly support the view that SNMP1 primarily functions as a high-sensitivity component of olfactory signal transduction and not as a specific element that is crucial for responding to a particular pheromone substance.

## 4. Materials and Methods

### 4.1. Animals

Desert locusts (*Schistocerca gregaria*) were reared (in the gregarious phase) as reported previously using metal cages (50 cm × 50 cm × 50 cm) and a light cycle of 12L:12D [81,82]. The temperature was 34 °C during the day and 27 °C at night. Fresh wheat seedlings and oat flakes were used to feed the locusts. The mutant SNMP1-deficient (SNMP1^−/−^) line originated in our own laboratory, and its generation has been described previously [26].

### 4.2. Preparation of Pipettes for Stimulus Delivery in EAG Recordings

Pheromonal compounds, plant odorants, and other odorous substances of high purity were purchased from Sigma-Aldrich (St. Louis, MO, USA) or TCI (Tokyo, Japan) and dissolved in dimethyl sulfoxide (DMSO; purity ≥ 99.5%; Sigma-Aldrich). For stimulation, glass Pasteur pipettes (150 mm; Neolab Migge, Heidelberg, Germany) were prepared as described previously [26]. Briefly, pipettes were equipped with a round piece of Whatman 40 filter paper (Sigma-Aldrich) that was loaded with 10 µL of the solvent DMSO or 10 µL of a 1:100, 1:1000 or 1:10,000 dilution of the relevant pheromone compound or odorant in DMSO and sealed with Parafilm M (Sigma-Aldrich). For linalool, a mixture of the two enantiomers [(±)-3,7-dimethyl-1,6-octadien-3-ol] was used (Sigma-Aldrich catalog number: L2602).

### 4.3. EAG Recordings

EAG experiments were conducted as detailed recently [26]. In brief, right antennae of sexually mature male desert locusts were excised and inserted between to glass micropipette electrodes. A humidified and constant airstream with a flow rate of 7 mL/s was directed towards the antenna through a mixing tube. For stimulation, the Parafilm sealing was removed from both sides of the glass Pasteur pipette containing a filter paper loaded with the (dilution of the) test substance or the solvent. The Pasteur pipette was connected to a CS 01 stimulus controller (Syntech, Kirchzarten, Germany) and the tip immediately inserted into a hole of the mixing tube to apply a 500 ms single puff into the airstream. Each antenna was stimulated twice by the test substance or the solvent. The order of the stimuli was changed every few recordings to minimize systemic errors. EAG amplitudes were recorded using the Syntech EAG software (version 2.7) and following the manufacturer’s advice (https://www.ockenfels-syntech.com/wp-content/uploads/EAGpract_man_fin; accessed on 30 August 2026). Raw data were exported to Microsoft Excel for further analysis. For each antenna, the value for the amplitude induced by the DMSO was subtracted from the amplitude values evoked by the respective pheromone or odorant. With the resulting values, for each compound, the mean was calculated based on the two technical replications. To assess for statistical significance, two-tailed *p*-values were calculated with an unpaired *t*-test (https://www.graphpad.com/quickcalcs/ttest1/, accessed on 30 August 2026). Statistical significance was set at *p* < 0.05 (**, 0.001 ≤ *p* < 0.01; *, 0.01 ≤ *p* < 0.05).

### 4.4. RNA Isolation, cDNA Synthesis and qPCR

The isolation of poly(A)^+^ RNA from 20 antennae of adult *S. gregaria* WT or SNMP1^−^/^−^ males as well as the subsequent synthesis of antennal cDNA were conducted as described previously [26].

In qPCR experiments, DNA sequences coding for OR3 (GenBank: KY964920.1), OR85 (GenBank: KY965002.1), OR96 (GenBank: KY965013.1), and the housekeeping gene glyceraldehyde-3-phosphate dehydrogenase (GAPDH; GenBank: HQ851387.1) were amplified. For amplification of OR-encoding sequences, primer pairs specific for OR3 (5′-gcactgcgtccagtaccacgacatg and 5′-gaatatggccagtacgaggtacgtc), OR85 (5′-tatcggcgttggatttttcg and 5′-atctggccgtaaaagtggac), and OR96 (5′-agcatggagaaccaactgcg and 5′-tacaggaacacaagtccgcc) were utilized. The primer pair 5′-attgttgaaggtctgatgacaacag and 5′-tccagttgatgctggaataatgttc was used to amplify a sequence coding for GAPDH, a reference gene verified for the normalization of expression in tissues from adult *S. gregaria* [83].

The qPCR reactions were prepared as detailed earlier [26] and run in duplicate on a Quantstudio 3 real-time PCR system (Thermo Fisher Scientific, Waltham, MA, USA) utilizing the following thermal cycling profile: 50 °C (for 2 min), 95 °C (for 2 min), followed by 40 steps of 95 °C for 15 s, 57 °C for 15 s, and 72 °C for 1 min. Melting curve analyses were carried out by running samples with the dissociation protocol (95 °C for 1 min, 60 °C for 1 min, and 95 °C for 15 s). Raw data were recorded and analyzed with the Quantstudio design and analysis software v1.5.1 (Thermo Fisher Scientific) and were exported to Microsoft Excel for further evaluation.

Using different primer concentrations, standard curves for the gene transcripts were generated in initial approaches with serial (10×) dilutions of the template. In these experiments, distinct primer concentrations were observed to allow optimal values for PCR efficiency (E) and the coefficient of determination (R^2^). For OR3, both primers were added at a concentration of 300 nM. For OR85 and OR96 both primers were used at a concentration of 500 nM, and for GAPDH, the concentration of both primers was 200 nM.

With the CT (threshold cycle) values determined in qPCR experiments, gene expression fold changes (relative expression) were calculated according to Pfaffl [84]. Two-tailed *p*-values for the relative expression were determined using an unpaired *t*-test (https://www.graphpad.com/quickcalcs/ttest1/, accessed on 30 August 2026). For the *t*-test, the values of the relative expression were compared to 1 (i.e., no change in expression). Statistical significance was set at *p* < 0.05.

### 4.5. Dual-Choice Olfactometer Behavioral Test

An airflow olfactometer (Figure S3) was utilized to investigate preference for wheatgrass (food plant) scents in desert locust nymphs about 20 days after hatching from the egg (nymph stages 3–4). To assess preferences for the odors of food plants, nymphs were used because, in our rearing conditions, feeding behavior is more pronounced in nymphs than in adults. For both genotypes (WT and SNMP1^−/−^), about half of the animals used were male; the rest were female. Prior to the experiment, the nymphs were kept individually and without food for about 24 h. The olfactometer test was carried out—with minor modifications—in a similar manner as described in a previous report with regard to setup and equipment [85]. In more detail, a cuboid observation chamber (60 cm × 30 cm × 30 cm) served as the test arena in which locust behavior was recorded. At the top, each half of the chamber was covered by a plastic plate with circular perforations (1 mm in diameter at 1 cm distance from each other). Both plates were equipped with an electrical fan (F12, 120 mm; Arctic, Braunschweig, Germany), each mounted in the center of the plate to direct the air upwards out of the chamber. In addition, two LED panels (daylight white, 6100 K, 120° beam angle, 2584 lumen, 15 W, 50 cm × 3 cm; LED-Leisten.com, Delitzsch, Germany) were mounted on the ceiling of the observation chamber. At the bottom of the arena, perforated plastic plates (perforation of 1 mm in diameter at 1 cm distance from each other) served as the floor for the locusts to walk on. Two plastic containers (28 cm × 28 cm × 18 cm) with open tops were put directly below the arena. The bottom of each container was connected to air tubes delivering air compressed by a silent compressor (850 W-Flüsterkompressor; Implotex, Wurmberg, Germany). Flowmeters (FR2000; Key Instruments by Brooks Instrument, Hatfield, PA, USA) were used to set a constant airflow at a rate of 1.75 L/min through each plastic container. 250-mL glass bottles (DWK Life Sciences, Wertheim, Germany) were placed between the flowmeters and the plastic containers to mix the airflows with the scent of chopped wheatgrass (approximately 50 mL, which corresponds to a net weight of 5–6 g), or they were left empty to provide a control stimulus (air). The entire setup was placed inside a larger acrylic box (70 cm × 55 cm × 90 cm), on top of which a funnel was mounted upside down to allow air to be exhausted by a radial fan (SPV Fan 100 T; Dalap, Olbernhau, Germany). The arena was covered with black tape, and the acrylic box was shielded from outside light with an opaque film. At the beginning of each behavioral test, a single locust entered the arena through a small tube in the middle of the observation chamber and the behavior in the olfactometer was recorded for the next 600 s by a camera (ELP-USBFHD05MT-KL36IR; Shenzhen Ailipu Technology Co., Shenzhen, China) placed at the ceiling of the arena. The temperature during the behavioral experiments ranged between 29 and 30 °C. On each test day, after testing half of the animals, the positions of the wheatgrass scent and the control (air) were reversed to avoid any potential positional effects. After testing a few animals, the arena was cleaned using 75% ethanol and ventilated for some time to remove potential odor residues. The wheatgrass was replaced with freshly cut wheatgrass after 2–3 animals to ensure a constant scent as a stimulus. In total, 22 WT and 22 SNMP1^−/−^ animals were recorded. For analysis, the total time an individual locust spent on each side of the arena was measured. Paired *t*-tests were applied to check for locust preference for a specific zone (wheatgrass or air). Statistical significance was set at *p* < 0.05 (***, *p* < 0.001; n.s., no statistical significance).

## Data Availability

The data supporting the findings of this study are included in this paper and its Supplementary Information. The raw data and experimental materials are available from the authors upon reasonable request.

## Supplementary Materials

The following supporting information can be downloaded at: https://www.mdpi.com/article/10.3390/ijms27188291/s1.

## Abbreviations

The following abbreviations are used in this manuscript:

cVA	11-cis-vaccenyl acetate
DMSO	dimethyl sulfoxide
EAG	electroantennogram/electroantennography
GAPDH	glyceraldehyde-3-phosphate dehydrogenase
ORCO	odorant receptor co-receptor
ORs	odorant receptors
OSNs	olfactory sensory neurons
PAN	phenylacetonitrile
qPCR	quantitative polymerase chain reaction
SEM	standard error of the mean
SNMP1	sensory neuron membrane protein 1
SNMP1^−/−^	SNMP1-deficient desert locust
VOCs	volatile organic compounds
WT	wild-type
