# Supplementary material for: SNMP1 Contributes to Sensitive Pheromone and Plant Volatile Detection in the Locust Olfactory System
Source: Int J Mol Sci. 2026 Sep 17;27(18):8291. doi: 10.3390/ijms27188291 (PMC13607538; doi:10.3390/ijms27188291)
Supplement: Supplementary file 1 [file ijms-27-08291-s001.zip › ijms-4473017-supplementary.pdf]

Figure S1

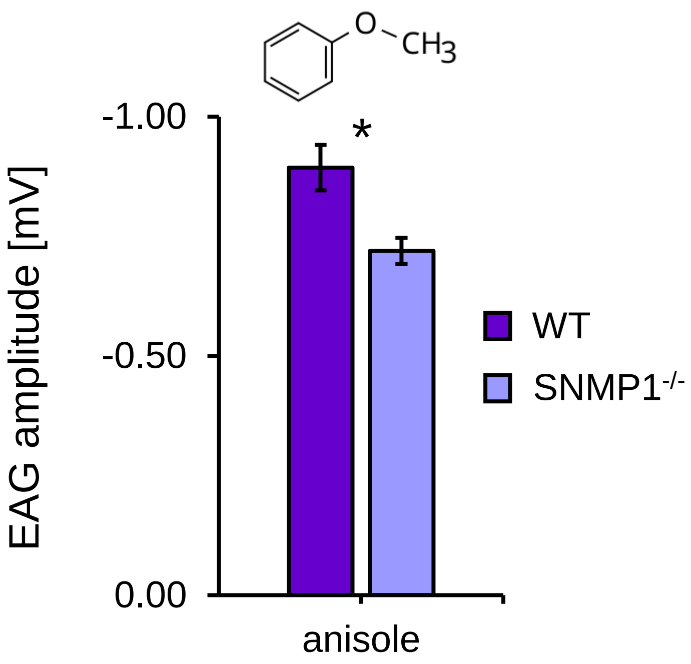

Figure S1. Antennal reactivity to the benzenoid substance anisole is diminished in SNMP1$^{-/-}$ desert locusts. Mean EAG amplitudes of WT (n=12) and SNMP1-deficient (n=13) males induced by stimulation with anisole are depicted. For each individual, only the right antenna was recorded. Error bars denote SEM. Two-tailed p-values were determined by an unpaired t-test (*, $0.01 \leq p < 0.05$).

Figure S2

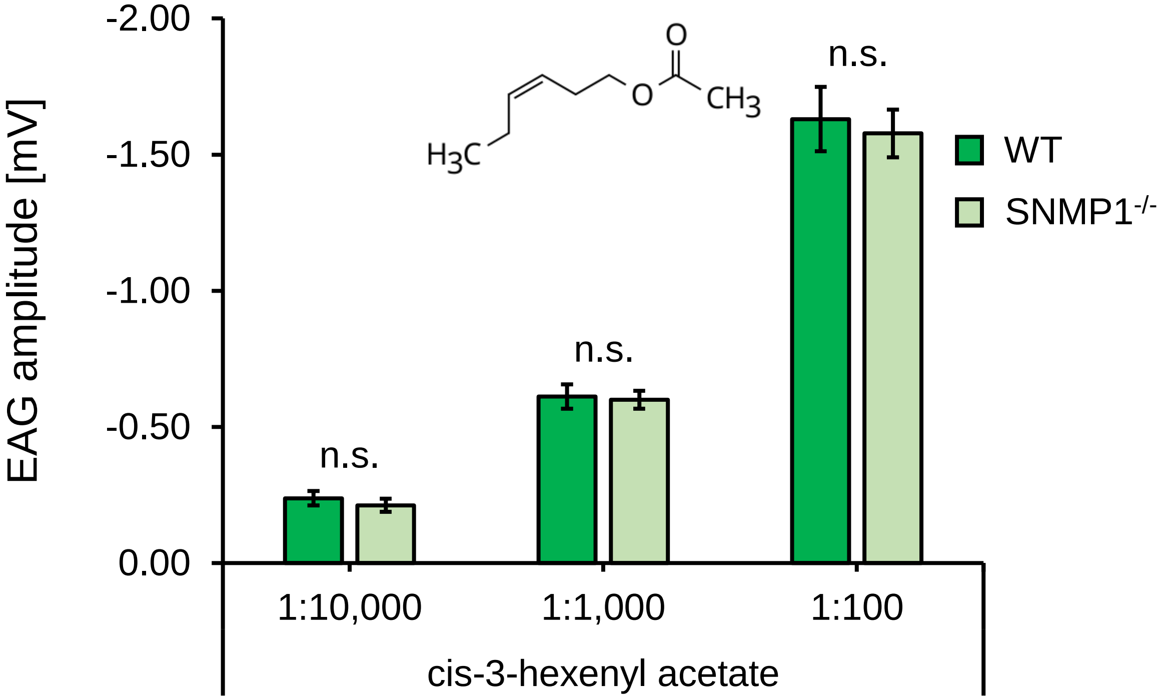

Figure S2. SNMP1 does not seem to contribute to the detection of the fatty acid-derived plant ester cis-3-hexenyl acetate. Mean EAG amplitudes induced by different dilutions of cis-3-hexenyl acetate in the right antenna of WT (n=12-13) and SNMP1$^{-/-}$ (n=11-12) males are depicted. The SEM is denoted by error bars and two-tailed p-values were determined by unpaired t-tests (n.s., no statistical significance).

Figure S3

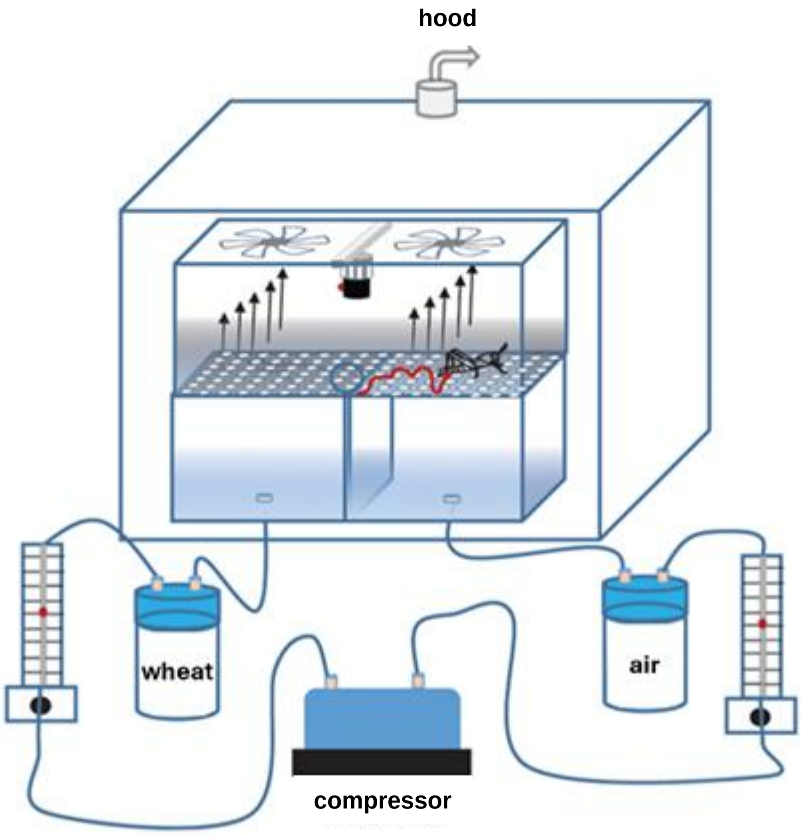

Figure S3. Schematic representation of the experimental setup used for the dual-choice olfactometer test. The olfactometer experiment was conducted as described in detail in the materials and methods section. The illustration was adapted from Chang et al. (2023) [85] with kind permission from Science.
